# A Comprehensive Coexpression Network Analysis in Vibrio cholerae

**DOI:** 10.1128/mSystems.00550-20

**Published:** 2020-07-07

**Authors:** Cory D. DuPai, Claus O. Wilke, Bryan W. Davies

**Affiliations:** a Institute for Cellular and Molecular Biology, University of Texas at Austin, Austin, Texas, USA; b Department of Integrative Biology, University of Texas at Austin, Austin, Texas, USA; c Department of Molecular Biosciences, University of Texas at Austin, Austin, Texas, USA; University of California, San Francisco

**Keywords:** *Vibrio cholerae*, computational biology

## Abstract

Research into the evolution and pathogenesis of Vibrio cholerae has benefited greatly from the generation of high-throughput sequencing data to drive molecular analyses. The steady accumulation of these data sets now provides a unique opportunity for *in silico* hypothesis generation via coexpression analysis. Here, we leverage all published V. cholerae RNA sequencing data, in combination with select data from other platforms, to generate a gene coexpression network that validates known gene interactions and identifies novel genetic partners across the entire V. cholerae genome. This network provides direct insights into genes influencing pathogenicity, metabolism, and transcriptional regulation, further clarifies results from previous sequencing experiments in V. cholerae (e.g., transposon insertion sequencing [Tn-seq] and chromatin immunoprecipitation sequencing [ChIP-seq]), and expands upon microarray-based findings in related Gram-negative bacteria.

**IMPORTANCE** Cholera is a devastating illness that kills tens of thousands of people annually. Vibrio cholerae, the causative agent of cholera, is an important model organism to investigate both bacterial pathogenesis and the impact of horizontal gene transfer on the emergence and dissemination of new virulent strains. Despite the importance of this pathogen, roughly one-third of V. cholerae genes are functionally unannotated, leaving large gaps in our understanding of this microbe. Through coexpression network analysis of existing RNA sequencing data, this work develops an approach to uncover novel gene-gene relationships and contextualize genes with no known function, which will advance our understanding of V. cholerae virulence and evolution.

## Abstract (precis)

Cholera is a devastating illness that kills tens of thousands of people annually. Vibrio cholerae, the causative agent of cholera, is an important model organism to investigate both bacterial pathogenesis and the impact of horizontal gene transfer on the emergence and dissemination of new virulent strains. Despite the importance of this pathogen, roughly one-third of V. cholerae genes are functionally unannotated, leaving large gaps in our understanding of this microbe. Through coexpression network analysis of existing RNA sequencing data, this work develops an approach to uncover novel gene-gene relationships and contextualize genes with no known function, which will advance our understanding of V. cholerae virulence and evolution.

## INTRODUCTION

Since the completion of the first Vibrio cholerae genome sequence in 2000, over 1,000 V. cholerae isolates have been sequenced (1, 2). These sequences have allowed for the development of sophisticated phylogeographic models, which emphasize the importance of controlling the spread of virulent and antibiotic-resistant V. cholerae strains to lower disease burden, in addition to fighting endemic local strains (2–6). The integration of hundreds of genomes paired with temporal and geographic information into ever-growing phylogenies enables analyses using selection models to predict future population trends and derive biologically meaningful insights into V. cholerae evolution (7, 8). By developing treatment and vaccination strategies based on phylogenetic models (9), organizations and governments can more efficiently leverage limited resources and more effectively prevent disease spread in line with the World Health Organization’s goal of eradicating cholera by 2030 (10).

Alongside advances in genomics research, the V. cholerae and broader bacterial biology communities have benefited greatly from other next-generation sequencing (NGS) technologies. Targeted sequencing experiments have been essential in mapping complex virulence pathways, illuminating a novel interbacterial defense system, and expanding our knowledge of the role of noncoding RNAs (ncRNAs) in the *Vibrio* life cycle (11–17). Further discoveries, such as transcription factor-mediated transposon insertion bias (18) and the role of cAMP receptor protein in host colonization (19), have benefited from composite research strategies utilizing multiple technologies. Similarly, meta-analyses utilizing pooled data from multiple experiments are empowered by the increasing availability of high-quality bacterial NGS data sets. Expression data are particularly amenable to such pooling and can be used to accurately group genes into functional modules based on their coexpression (20). In bacteria, weighted gene coexpression network analysis (WGCNA) (21) has been successfully used to underscore biologically important genes and gene-gene relationships via “guilt-by-association” approaches (22, 23). These studies have taken advantage of larger and larger heterogeneous microarray data sets to provide novel biological insights via existing data.

Despite major advances in sequencing technologies and research strategies, most of the over two dozen existing transcriptome sequencing (RNA-seq) experiments in V. cholerae have been limited to targeted approaches that involve quantifying the differential abundance of genetic material across a few conditions. Via these approaches, it is nearly impossible to generalize about any change in expression observed in one experiment to other treatment conditions, and analyses are limited to a few pathways or genes of interest. In contrast, meta-analyses such as WGCNA can uncover much broader relationships throughout the genome by combining information from multiple data sets. As there is no existing coexpression analysis in V. cholerae to date, the accumulation of over 300 publicly available RNA-seq samples from targeted RNA-seq experiments represents a heretofore untapped resource for the cholera community.

Motivated by the success of pooled genetic sequencing analyses, our current work utilizes all publicly available V. cholerae RNA-seq-based expression-level data to generate a coexpression network. We expand upon existing bacterial WGCNA approaches by integrating broader sequencing data (including chromatin immunoprecipitation sequencing [ChIP-seq] and transposon insertion sequencing [Tn-seq]) and multiple annotation platforms into our analysis. Our network ultimately contributes information on connections across all V. cholerae genes, including the roughly 1,500 predicted but functionally unannotated genetic elements that account for some 37% of the genome. More specifically, we implicate new loci in virulence regulation and clearly demonstrate a powerful and accurate approach to hypothesis generation via our described network.

## RESULTS

### Gene network generation.

To generate our coexpression analysis in V. cholerae, we applied our WGCNA pipeline to analyze 27 V. cholerae RNA sequencing experiments deposited in NCBI’s Sequence Read Archive (SRA) in addition to two novel experiments. The RNA sequencing samples are derived from experiments exploring a range of important V. cholerae processes including intestinal colonization, quorum sensing, and stress response. In total, our network includes 300 individual RNA-seq samples (see Table S1 in the supplemental material). All samples were mapped to a recently inferred V. cholerae transcriptome derived from the N16961 reference genome (1, 13). This reference was chosen because the majority (293) of samples were collected from strain N16961 or the closely related strains C6706 and A1552.

10.1128/mSystems.00550-20.3 TABLE S1 Data sets included in network analysis. This table indicates the SRR numbers (filename) and SRP numbers (SRA_study) of all RNA-seq data used in network construction. Download Table S1, XLSX file, 0.02 MB. Copyright © 2020 DuPai et al. 2020 DuPai et al. This content is distributed under the terms of the Creative Commons Attribution 4.0 International license.

**FIG 1:**
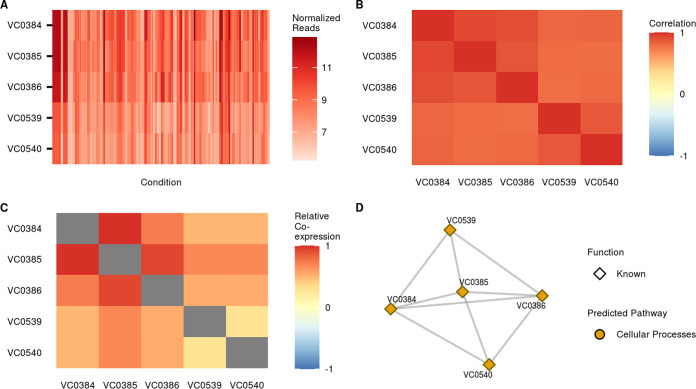
General outline of network construction. To explain the overall WGCNA process, we have chosen a subset of genes that are involved in the same core process, cysteine metabolism. Loci VC0394 to VC0386 are predicted to fall within one operon while loci VC0539 and VC0540 are predicted to be in another. (A) Normalized (log_2_) expression reads for the same genes across multiple conditions supply the basis for our coexpression analysis. (B) Correlations are calculated from the normalized counts shown in panel A for every pair of genes. (C) An adjacency matrix (not shown) was calculated from the correlations shown in panel B and ultimately used to produce a topological overlap matrix (TOM) that supplies network edge weights with less noise than the raw correlation matrix. While the signal of coexpressing pairs is dampened slightly, this step greatly decreases spurious relationships as it favors transcripts which coexpress with similar sets of genes rather than potentially noisy direct correlations. (D) The final network groups transcripts that tightly coexpress while indicating what pathways they are involved in. In this example, all genes significantly coexpress, with the exception of VC0539 and VC0540 despite their colocalization within the same operon. After network construction, information was added to label genes based on their function and essentiality under virulence and growth conditions.

Figure 1 outlines the process used to generate our coexpression network with a small subset of genes. The five included loci are known to be involved in cysteine metabolism with loci VC0384 to VC0386 and loci VC0539 and VC0540 falling within two separate operons. Following normalization of mapped transcripts (Fig. 1A), a weighted gene coexpression network analysis was performed using WGCNA, as follows (21). First, a Pearson correlation matrix was calculated for expression levels of all genes (Fig. 1B). This correlation matrix clearly captures strong relationships between coexpressing genes but can produce background noise from unrelated gene pairs and underlying gene structures (i.e., operons). We limit this noise by calculating a topological overlap matrix (TOM) (24) that weights pairwise coexpression data based on each gene’s interactions with all other genes (Fig. 1C). In this way, the relationships between genes that fall within the same subnetwork are favored while signals from less tightly coregulated genes are abated. This TOM, after filtering is performed for normalized values greater than 0.1, is used to construct an accurate coexpression network that captures biologically meaningful relationships while minimizing background noise (Fig. 1D).

In addition to coexpression data, our network and analyses incorporated information from multiple other sources. Our network includes predicted pathway annotations and gene functional knowledge from the NCBI Biosystems database as well as the DAVID, Panther, and KEGG databases (25–28). Operon structure was inferred using Operon-mapper (29). Additionally, importance labels were applied to genes with no known function which have been implicated as playing a role in intestinal colonization or *in vitro* growth via *Tn*-seq-based essentiality experiments (14, 30). Information from ChIP-seq binding assays and microarray results were incorporated in downstream analyses to substantiate network-derived relationships. By combining all of these data sources, we were able to develop and analyze an informative network of coexpressing genes that provides both qualitative and quantitative information about relationships between transcripts across 49 gene clusters covering the entire V. cholerae genome (Data Set S1 and S2).

10.1128/mSystems.00550-20.6 DATA SET S1 Gene coexpression network. The full coexpression network file is accessible via Cytoscape and similar software. Download Data Set S1, TXT file, 84.4 MB. Copyright © 2020 DuPai et al. 2020 DuPai et al. This content is distributed under the terms of the Creative Commons Attribution 4.0 International license.

10.1128/mSystems.00550-20.7 DATA SET S2 Gene coexpression network labels. Labels are for the network reported in Data Set S1. Download Data Set S2, TXT file, 0.1 MB. Copyright © 2020 DuPai et al. 2020 DuPai et al. This content is distributed under the terms of the Creative Commons Attribution 4.0 International license.

### A network of novel, unexpected, and informative interactions.

**FIG 2:**
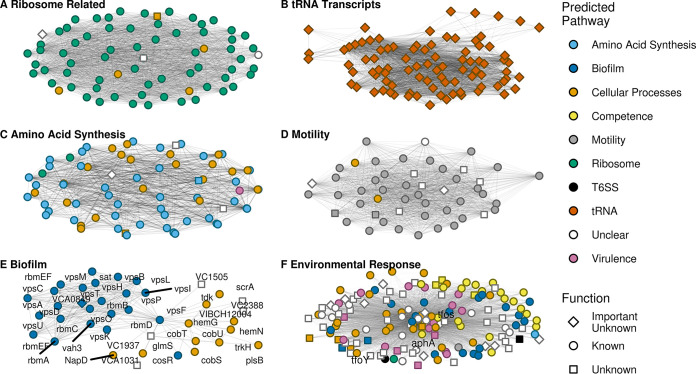
Subnetworks recapitulating known results. The depicted subnetworks each contain transcripts that are known to be largely involved in one or more related biological process(es). For each subnetwork, the nodes represent transcripts while the edges represent a coexpression relationship of at least 0.1 between transcripts. (A to F) Subnetworks involved in the following core processes: ribosome related, tRNA transcripts, amino acid synthesis, motility, biofilm, and environmental response.

As many functionally related bacterial genes are coexpressed in operons such as the operon of VC0384 to VC0386 above, we sought to discover if operon structure was a contributing factor to our network or specific subnetworks. Indeed, gene pairs predicted to fall within the same operon did show significantly higher average normalized coexpression than their nonoperon counterparts (0.186 versus 0.147; *P* < 0.001), and some subnetworks, such as the ribosome-related subnetwork (Fig. 2A), contain a high proportion of intraoperon gene pairs (Fig. S1). However, across our full network only 0.2% of all coexpressing gene pairs fell within the same operon, and no subnetwork had a majority of such pairs (Fig. S1). Moreover, our overall network captured information on relationships with roughly one-third of unannotated V. cholerae genes (Fig. S2), providing insight into functional roles that are not obvious based on gene homology or known operon structure.

10.1128/mSystems.00550-20.1 FIG S1 Operon relationships between coexpressing genes. Each graph illustrates the percentage of coexpressing gene pairs within a given network or subnetwork that are predicted to be within the same operon (red), not within the same operon (black), or that lack operon prediction data (grey). Networks and subnetworks mentioned in detail in this text are highlighted in orange. Download FIG S1, TIF file, 0.3 MB. Copyright © 2020 DuPai et al. 2020 DuPai et al. This content is distributed under the terms of the Creative Commons Attribution 4.0 International license.

10.1128/mSystems.00550-20.2 FIG S2 Functional knowledge of network genes. Each graph illustrates the percentage of known (blue), unknown (red), and important unknown (pink) genes within a given network or subnetwork. Important unknown refers to genes that are essential or semiessential for growth or intestinal colonization (14, 30). Networks and subnetworks mentioned in detail in this text are highlighted in orange. Download FIG S2, TIF file, 0.3 MB. Copyright © 2020 DuPai et al. 2020 DuPai et al. This content is distributed under the terms of the Creative Commons Attribution 4.0 International license.

### Genes in known pathways cluster together and contextualize genes of unknown function.

**FIG 3:**
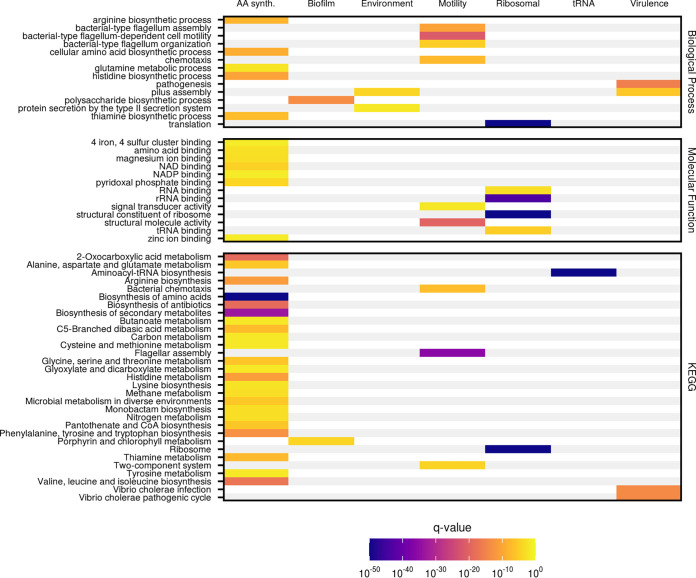
Significantly enriched GO and KEGG terms for specific subnetworks. The indicated terms are significantly enriched within highlighted pathways, with the color indicating the significance of the enrichment as determined via the false-discovery rate-adjusted *P* value (*q* value). The terms are divided by database and, for Gene Ontology (GO) terms, GO domain, as indicated to the right.

As a demonstration of the accuracy of our approach, we have highlighted several clusters that recapitulate known interactions between transcripts involved in highly conserved, well-studied cellular processes (Fig. 2). The correct grouping of transcripts encoding ribosomal proteins, tRNAs, and amino acid synthesis proteins into significantly coexpressing subnetworks provided a positive control for our overall network (Fig. 2A to C). Importantly, our analysis clustered together genes known to be involved in more specialized processes such as motility and biofilm formation (Fig. 2D and E), with corresponding Gene Ontology (GO) (31) and KEGG (27) pathway terms enriched for genes within these subnetworks (Fig. 3 and Table S2).

10.1128/mSystems.00550-20.4 TABLE S2 Significantly enriched GO and KEGG terms. Significantly enriched Gene Ontology (GO) and KEGG Pathway terms are listed for all subnetworks within our larger network. Download Table S2, XLSX file, 0.03 MB. Copyright © 2020 DuPai et al. 2020 DuPai et al. This content is distributed under the terms of the Creative Commons Attribution 4.0 International license.

In addition to capturing relationships between genes involved in specific pathways, our approach can also accurately group genes involved in interconnected processes that share overlapping regulation, as seen in the environmental sensing subnetwork (Fig. 2F). This subnetwork includes high-level transcriptional regulators, such as AphA, TfoS, and TfoY, with known roles mediating the complex interplay between quorum sensing, natural competence, type VI secretion, and other related pathways (32–37). As each of these transcription factors is involved in a multitude of cellular processes and significantly coexpresses with hundreds of other genes, our analysis describes their closest connections under parameters designed to find meaningful and practically interpretable relationships. By altering these parameters (significance cutoffs, minimum number of genes per cluster, clustering algorithm, etc.), analysis of the overall network can be fine-tuned to focus in on specific biological processes or explore the nodes that drive connections between processes that are necessary for V. cholerae to adapt and survive in diverse environments.

The subnetworks outlined in Fig. 2 support the utility of our analysis in powering the inference of gene function based on guilt by association (38). Because each of these gene clusters contains coexpressing genes that are involved in the same biological process, it can be assumed that unannotated genes in the same cluster are likely involved in the same process. Such links, while not definitive on their own, can be used with other data to hint at gene functions. For example, the genes with known function shown in Fig. 2E are primarily involved in biofilm formation (39, 40). This clustering of biofilm genes suggests that the few genes with no known function in this subnetwork may be involved in the same process. Two of these unannotated transcripts, VC1937 and VC2388, are, per GO cellular component location labels, integral membrane components. Further, the VC2388 locus is directly upstream of a Vcr084, a short RNA involved in quorum sensing which is essential for biofilm formation (41). Taken together, this evidence suggests that VC1937 and VC2388 may play a role in some of the complex membrane restructuring necessary for biofilm formation. In facilitating such guilt-by-association approaches to novel hypothesis generation, our coexpression network serves as a highly efficient substitute for more traditional screening assays.

### A virulence subnetwork suggests novel gene functions.

**FIG 4:**
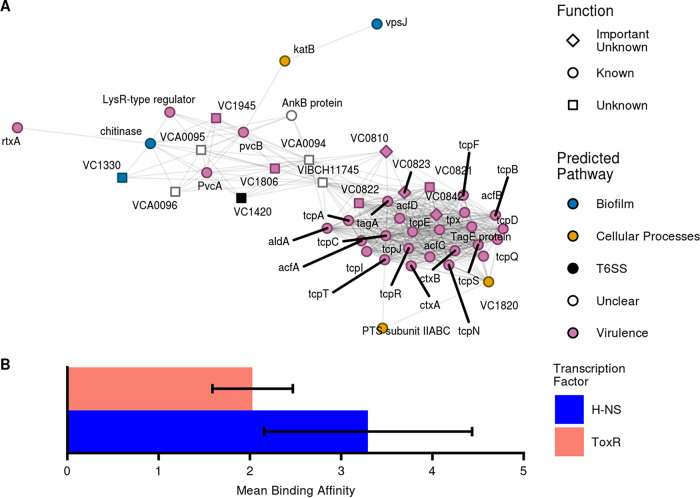
Virulence-related subnetwork. (A) This subnetwork contains a majority of genes that are predicted to be involved in virulence-related pathways, providing clues to the genes with no known functions, such as those at loci VCA0094 to VCA0096. PTS subunit IIABC, phosphotransferase system fructose-specific transporter subunit IIABC; T6SS, type 6 secretion system. (B) Mean binding affinity (log_2_ fold change in occupancy compared to level of the loading control) for different virulence-associated transcription factors near loci VCA0094 to VCA0096. Both H-NS and ToxR show significant binding preferences for this region. Error bars indicate standard deviations from the means.

While the biofilm-associated subnetwork (Fig. 2E) presents a relatively simple example of the functional insights our coexpression data can yield, the virulence-related subnetwork (Fig. 4A) represents a more complex case in which genes of known function provide clues to the role of unannotated genes. The majority of transcripts in this module originate from within the virulence-related ToxR regulon that consists principally of genes on V. cholerae pathogenicity island 1 (VPI-1) (VC0809 to VC0848) and cholera toxin subunits A and B (*ctxAB*, VC1456, and VC1457) (42). Other genes in this subnetwork, such as *vpsJ*, VC1806, VC1810, and chitinase, are predominately localized to virulence islands and other areas of the genome under tight control of the known virulence regulator ToxR, ToxT, or H-NS as determined via ChIP-seq and/or RNA-seq (43–45). Genes in this subnetwork are also enriched for virulence-related GO and KEGG terms, such as “pathogenesis” and “Vibrio cholerae infection” (Fig. 3). The clustering of such genes with well-characterized interactions into a cohesive subnetwork is further validation of our ability to generate accurate coexpression maps of related genes. The association of uncharacterized genes in these clusters suggests that they may also play a role in V. cholerae virulence and generates hypotheses about the function of unknown genes within this module.

Many of the important transcripts with unknown function are expected to coexpress with known virulence genes because they fall within VPI-1 (VC0810, VC0821 to VC0823, and VC0842) or VPI-2 (VC1806 and VC1810) or are proximal to other virulence genes (VC1945) (46, 47). However, our analysis also identified genes such as VCA0094 to VCA0096 which are on a completely different chromosome than the rest of the subnetwork and do not neighbor any known virulence elements.

A major benefit of our approach is that we incorporate additional regulatory data such as ChIP-seq and Tn-seq into our coexpression analysis, allowing us to verify the association between VCA0094 to VCA0096 and virulence pathways using existing experimental data. Tn-seq analysis has previously identified VCA0094 and VCA0095 as essential for infection of a rabbit intestine (14), suggesting that these loci play a role in virulence. Because transcripts for these genes coexpress with genes regulated by ToxT, ToxR, and H-NS, we also probed existing ChIP-seq binding data sets (12, 19, 43) to see if any of these well-studied transcription factors bind near loci VCA0094 to VCA0096. While ToxT binding was not observed near this site (data not shown), our analysis identified significant peaks in the promoter region of VCA0094 for both ToxR and H-NS, as calculated via reanalysis of existing binding data from Kazi et al. (43). Both peaks showed large and significant increases in binding affinity (log_2_ fold change in average occupancy) compared to levels of the input controls (Fig. 4B). H-NS showed a clear binding peak in the region of the VCA0094 promoter that extended in a diffuse manner to the VCA0095 transcription start site while ToxR binding covered a similar region but was more diffuse throughout (data not shown). Collectively, these results indicate virulence-related functions for the products of the transcripts of VCA0094 to VCA0096. Although the exact mechanistic role of these genes remains elusive, we have nevertheless demonstrated the ability of our pipeline to generate meaningful hypotheses by incorporating existing data from a multitude of sources.

### Coexpression data provides an accurate *in silico* complement to RNA-seq.

In addition to the guilt-by-association inference described above, coexpression analysis can provide a partial substitute or complement to RNA-seq experiments. Novel, meaningful genetic relationships can be found in a coexpression network by focusing on the transcripts that are coregulated with a gene of interest.

**FIG 5:**
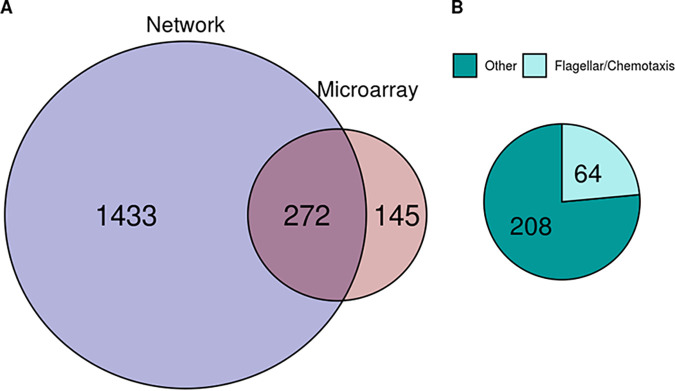
Comparing RpoS microarray data to data of coexpressing genes in our WGCNA. (A) Overlap of genes with expression patterns related to the pattern of *rpoS* expression as identified via our network analysis (blue) and existing microarray data (red). The overlapping region identifies 272 genes that are common between the two analyses. (B) Breakdown of shared genes (overlapping region in panel A). All of the flagellar and chemotaxis genes highlighted as particularly important in the microarray data set are identified by both methods.

We can apply a network-based approach in lieu of new RNA-seq-based experiments to identify genes which coexpress with *rpoS* (VC0534) and are similarly involved in the bacterial stress response. As our network utilizes only RNA-seq-based transcriptomics studies and as none of these studies involves direct manipulation of *rpoS*, we can compare existing microarray data involving an *rpoS* (VC0534) deletion mutant (48) to determine how accurate our approach is. When an absolute coexpression cutoff of 0.1 is applied, 272 genes are identified as having a relationship with *rpoS* expression in both our network analysis and the *rpoS* mutant microarray data (Fig. 5A). This represents nearly two-thirds of genes identified as differentially expressed in the original microarray study. While our network links far more genes with *rpoS* than the microarray approach, this is in line with recent RNA-seq-based work that found that 23% of the Escherichia coli genome is regulated by RpoS (49). Additionally, all of the flagellum- and chemotaxis-related proteins highlighted as particularly informative in the original study were identified by our analysis (Fig. 5B), and relevant values (i.e., network coexpression and microarray-derived log fold change in expression) for the 273 shared transcripts have a Spearman correlation of −0.516. This accuracy was achieved without any direct genetic manipulation of the *rpoS* locus in the RNA-seq data sets used to generate our coexpression network and serves as a testament to the potential utility and versatility of our approach.

Our approach to isolating genetic interactions also has advantages over transcriptomics-focused sequencing. As seen in Fig. 5A, our network-based analysis identified far more genes associated with *rpoS*. This is likely because RNA-seq-based approaches can identify a broader range of gene transcripts as they are not limited by restrictive microarray probes (50). Separate from differences in underlying technology, coexpression networks are also more likely to detect genes regulating a target’s expression than traditional transcriptomics experiments, which largely capture downstream responses to changes in a target’s expression (51, 52). Thus, a coexpression network can provide an alternative perspective to complement or clarify transcriptomics data.

## DISCUSSION

We have successfully constructed the first V. cholerae coexpression network through a computationally inexpensive process that is simple, easily expanded upon, and straightforward to implement in other organisms. Our network effectively identifies canonical gene clusters related to specific molecular pathways or functions, such as those corresponding to tRNAs or biofilm proteins. We have also outlined two use cases for the data provided and have shown the accuracy of both approaches using existing data. Additionally, we have included relevant network files as well as raw read counts across RNA-seq conditions (see Data Sets S1 and S2 and Table S3 in the supplemental material) alongside all code used in our analysis (see Materials and Methods) to encourage broad usage of these data.

10.1128/mSystems.00550-20.5 TABLE S3 Normalized read counts. Normalized read counts for all RNA-seq experiments used in network construction. Columns indicate experiments as labeled in Table S1; rows indicate normalized counts for the given locus. Download Table S3, XLSX file, 9.0 MB. Copyright © 2020 DuPai et al. 2020 DuPai et al. This content is distributed under the terms of the Creative Commons Attribution 4.0 International license.

Our results have proven both the utility and accuracy of our approach despite in-depth analysis limited to a few genes across 5 of the 49 observed gene clusters. Furthermore, our work with the virulence subnetwork supports previously published research loosely implicating genes VCA0094 to VCA0096 in virulence and virulence-related functions. All three transcripts have shown up in screens focusing on biofilm development (53) and the SOS response (13). From a mechanistic perspective, protein homology analysis via NCBI’s Conserved Domain Database (54) indicates that VCA0094 possesses a DNA-binding transcriptional regulator domain while VCA0096 contains domains that implicate it in protein activation via proteolysis. These data combined with our novel findings hint at the potential biological importance of this genomic locus.

When viewed through the lens of a specific gene of interest, coexpression data are in large part analogous to the differential expression data produced by RNA-seq experiments. While RNA-seq offers finer assay control and can be tailored more exactly to suit a specific research question, there are both technical and practical limitations that may make such an approach impractical. Whether an experimenter is interested in examining the role of an essential locus or is limited by available resources, our coexpression analysis presents a fast, free, and faithful alternative for probing genetic interactions, as outlined in our analysis of *rpoS* above.

Major motivations for this work include the successful implementation of bacterium-focused, microarray-based coexpression networks and the lack of clear functional knowledge for a large portion of V. cholerae genes. In addition to simpler guilt-by-association studies (22, 23), coexpression networks have helped to elucidate relationships in diverse microbial communities (55–58) and to enable comparisons across strains and species (59–61). These works as well as the relative dearth of knowledge about the V. cholerae genome (roughly two-thirds of genes are annotated whereas around 86% percent of all E. coli genes are annotated [62]) and the growing abundance of V. cholerae-focused NGS data served as the impetus for this research.

The calculated coexpression network, though accurate, could be improved via the inclusion of more experiments and more extensive SRA annotations. Our somewhat limited pooled data set consisting of 300 samples is an order of magnitude below the few thousand samples necessary to derive the most faithful coexpression estimates (63). Though sample size will improve as more V. cholerae RNA-seq experiments are published, more samples may also increase the risk posed by batch effects which cause spurious correlations among genes through technical variation (64, 65). The diverse structure of our current data helps to minimize the impact of batch effects, but this would be offset by the future inclusion of larger data sets from single experiments. While automated sample clustering methods (66–68) can effectively group overly correlated samples, there is no way to know if the correlation is biological (i.e., meaningful) or technical (i.e., noise) in origin. Similarly, manual curation of batch annotations is also difficult since few SRA records are extensively annotated with detailed experimental conditions (e.g., bacterial growth stage or exact medium used). Thus, careful consideration may be necessary when expanding and generalizing this analysis to include future data.

The mapping of raw reads to a transcriptome derived from a single reference genome presents a limitation to our current work. While this approach is reasonable given the similarity of the vast majority of included strains to our reference, a more elaborate comparative transcriptomic strategy (69, 70) would be ideal if more diverse samples are included in future analyses. This is especially true when we consider the inclusion of expression data from clinical samples which are likely to have much more genomic variability than the closely related lab cultured strains used to construct our network. On the other hand, because comparative transcriptomics requires defining homologous alleles across all strains analyzed (71), such an approach would greatly increase the difficulty of incorporating strains without an assembled genome.

In summary, our coexpression network can drive functional hypotheses for unannotated genes in V. cholerae. As the *Vibrio* community steadily adds high-quality data from increasingly sophisticated sequencing experiments to public databases, our imputed network can only improve, providing ever-deeper insights into the V. cholerae genome. At the same time, highly annotated transcript-based coexpression networks can empower research with related technologies (e.g., single-cell transcriptomics and dual RNA-seq) and research into a host of other clinically relevant bacteria, such as Pseudomonas aeruginosa or Staphylococcus aureus, for which there are over 2,000 and 1,400 RNA-seq experiments, respectively, in the SRA.

## MATERIALS AND METHODS

### Data collection and processing.

All RNA and ChIP sequencing data were downloaded from the Sequence Read Archive (SRA) (72) and converted to compressed fastq files using the SRA Toolkit (https://trace.ncbi.nlm.nih.gov/Traces/sra/sra.cgi?view=software) (see Table S1 in the supplemental material for details on included experiments). RNA-seq samples were selected by searching the SRA on 10 September 2019 for the organism and strategy terms “vibrio cholerae” and “rna seq,” respectively, resulting in 326 initial samples including the 34 novel samples from this publication . Samples were mapped to a recently inferred V. cholerae transcriptome derived from the N16961 reference genome (1, 13) using Kallisto, version 0.45.1 (73). This reference was chosen because the majority (293) of samples were collected from strain N16961 or the closely related C6706 and A1552 strains. Twenty-six low-quality samples with <50% of reads mapping to the reference transcriptome were discarded before further analysis, leaving 300 samples used for further analysis.

For ChIP-seq analysis, accession numbers were identified via the relevant publications (12, 19, 43), and sequences were downloaded from the SRA and converted to fastq files as described above. Raw reads were mapped to the same N16961 reference genome using Bowtie 2, version 2.3.5.1 (74). From this mapping, peaks were identified using MACS2, version 2.1.2, with the parameter extsize set at 225 (various sizes from 150 to 500 were tested with little observable difference in the peaks identified) (75), and differential binding and significance were calculated using DiffBind, version 2.12.0 (76).

Processed Tn-seq data were collected directly from published data sets. *In vitro* essentiality and semiessentiality labels were derived from Table S1 in Chao et al. (30), with the original labels of domain essential and sick genes replaced with essential and semiessential, respectively. We used Table S2 from Fu et al. (14) to label genes involved in host infection, with any gene exhibiting a log_2_ fold change of less than −3 deemed essential and any gene with a log_2_ fold change between −1 and −3 deemed semiessential.

### Network construction.

Figure 1 highlights the process used to generate our coexpression network. Kallisto-derived reads were first imported into R via tximport (77) and then normalized using DESeq2, version 1.24.0 (78), resulting in values that are comparable across conditions and experiments. Following normalization, a weighted gene coexpression network analysis was performed using WGCNA (21). This process is highlighted with a subset of data in Fig. 1 and consists of the sequential calculation of a Pearson correlation matrix, adjacency matrix with power β = 6, and, ultimately, a topological overlap matrix (TOM) (24) from normalized gene expression counts across conditions. We further filtered this TOM to exclude samples with weighted coexpression of <0.1 for all analyses included in Results.

Predicted pathway annotations and gene functional knowledge were derived from the NCBI Biosystems database as well as the DAVID, Panther, and KEGG databases (25–28). Genes for which functional knowledge is lacking and which are identified as essential or semiessential in either Tn-seq data set are labeled in network visualizations as “important unknown.” Operon predictions were inferred using Operon-mapper (29).

### Data availability.

Information on the 34 novel samples are available in the SRA database under accession numbers SRR10905341 to SRR10905344, SRR10905351, SRR10905362, and SRR10905369 to SRR10905396 and under BioProject accession number PRJNA601792. SRA accession numbers and information on all included samples can be found in Table S1 in the supplemental material. A full, unfiltered network graph is provided in Data Set S1, with the corresponding node labels given in Data Set S2. Raw, unnormalized read counts are also provided in Table S3. All data analysis and figure generation were performed using the R programming language, with code available at https://doi.org/10.5281/zenodo.3572870.
