# Supplementary figures and images for: A Comprehensive Coexpression Network Analysis in Vibrio cholerae
Source: mSystems. 2020 Jul 7;5(4):e00550-20. doi: 10.1128/mSystems.00550-20 (PMC7343309; doi:10.1128/mSystems.00550-20)

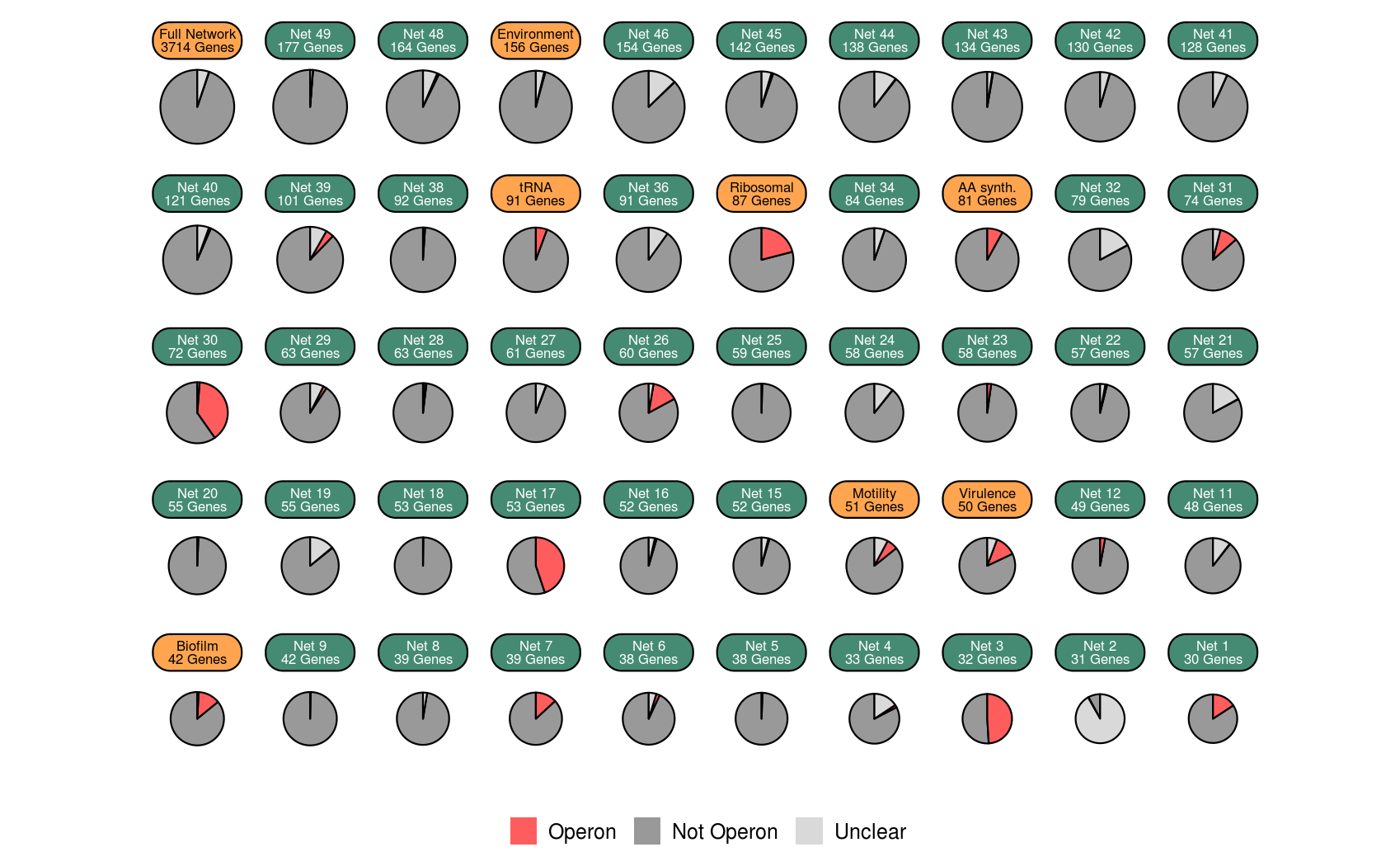

Supplement: FIG S1 [file mSystems.00550-20-sf001.tif]

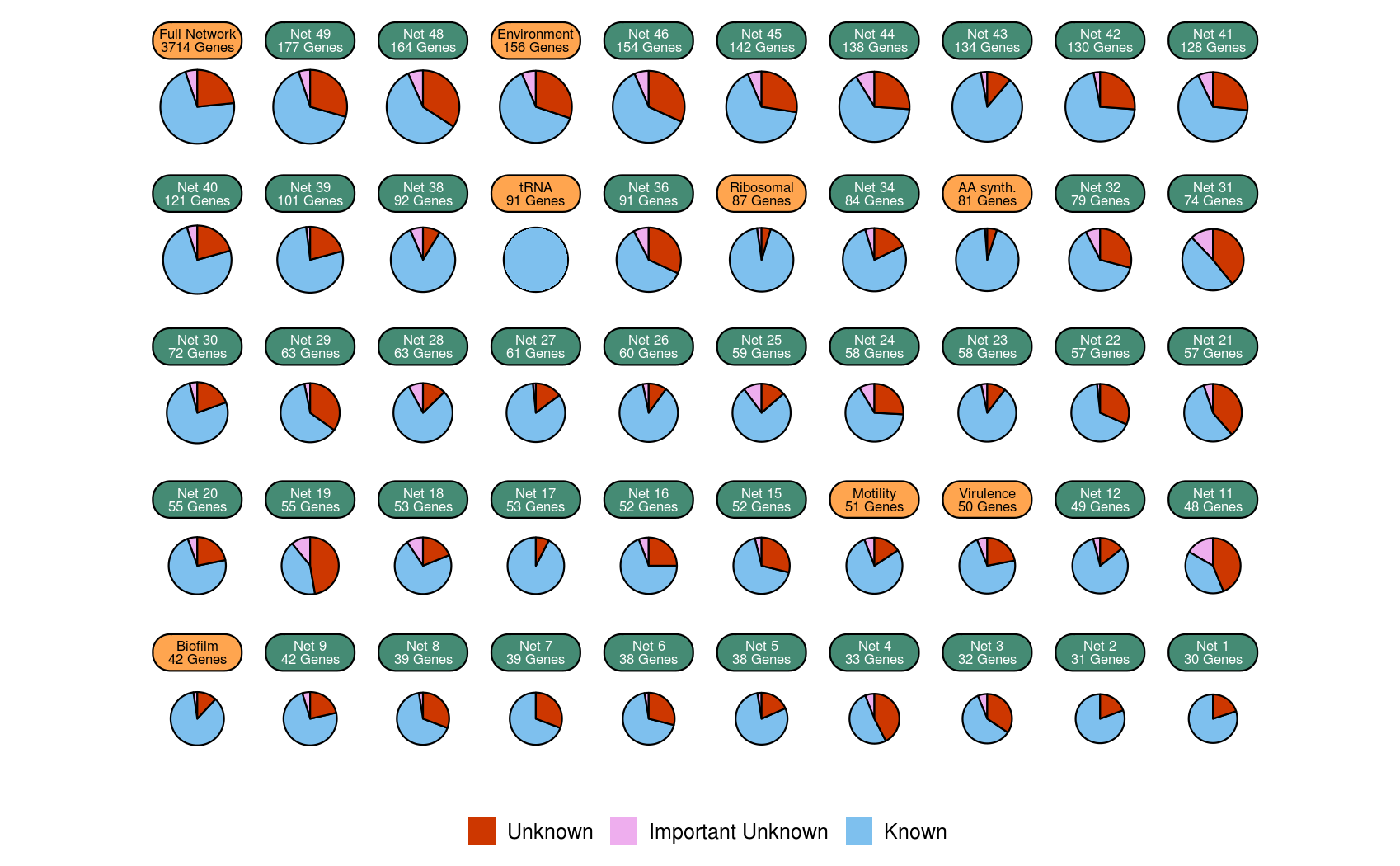

Supplement: FIG S2 [file mSystems.00550-20-sf002.tif]
